# High Density, Double-Sided, Flexible Optoelectronic Neural Probes With Embedded μLEDs

**DOI:** 10.3389/fnins.2019.00745

**Published:** 2019-08-09

**Authors:** Jay W. Reddy, Ibrahim Kimukin, Luke T. Stewart, Zabir Ahmed, Alison L. Barth, Elias Towe, Maysamreza Chamanzar

**Affiliations:** ^1^Department of Electrical and Computer Engineering, Carnegie Mellon University, Pittsburgh, PA, United States; ^2^Department of Biological Sciences, Carnegie Mellon University, Pittsburgh, PA, United States; ^3^Carnegie Mellon Neuroscience Institute, Carnegie Mellon University, Pittsburgh, PA, United States

**Keywords:** neural probe, micro-LED, Parylene C, microfabrication, optogenetics

## Abstract

Optical stimulation and imaging of neurons deep in the brain require implantable optical neural probes. External optical access to deeper regions of the brain is limited by scattering and absorption of light as it propagates through tissue. Implantable optoelectronic probes capable of high-resolution light delivery and high-density neural recording are needed for closed-loop manipulation of neural circuits. Micro-light-emitting diodes (μLEDs) have been used for optical stimulation, but predominantly on rigid silicon or sapphire substrates. Flexible polymer neural probes would be preferable for chronic applications since they cause less damage to brain tissue. Flexible μLED neural probes have been recently implemented by flip-chip bonding of commercially available μLED chips onto flexible substrates. Here, we demonstrate a monolithic design for flexible optoelectronic neural interfaces with embedded gallium nitride μLEDs that can be microfabricated at wafer-scale. Parylene C is used as the substrate and insulator due to its biocompatibility, compliance, and optical transparency. We demonstrate one-dimensional and two-dimensional individually-addressable μLED arrays. Our μLEDs have sizes as small as 22 × 22 μm in arrays of up to 32 μLEDs per probe shank. These devices emit blue light at a wavelength of 445 nm, suitable for stimulation of channelrhodopsin-2, with output powers greater than 200 μW at 2 mA. Our flexible optoelectronic probes are double-sided and can illuminate brain tissue from both sides. Recording electrodes are co-fabricated with μLEDs on the front- and backside of the optoelectronic probes for electrophysiology recording of neuronal activity from the volumes of tissue on the front- and backside simultaneously with bi-directional optical stimulation.

## 1. Introduction

Optical methods have been widely used for stimulation and functional imaging of neural circuits (Delbeke et al., 2017; Yang and Yuste, 2017). In particular, optogenetics has been used as a powerful tool for selective excitation or inhibition of specific cell types using light of different wavelengths (Chen et al., 2017; Zhao, 2017). To isolate details of neural circuit functions at different stages of health and disease, it is desirable to stimulate or inhibit a subset of neurons that express the same light-sensitive proteins. This requires delivering patterns of light into brain tissue with high spatial resolution. Conventional methods based on external optics for light delivery are limited to superficial layers of the tissue because of absorption and scattering (Ntziachristos, 2010). Moreover, in the context of closed-loop optogenetic experiments, recording electrodes must be integrated on an optical neural probe to enable simultaneous electrical recording and optical stimulation. Recently, optoelectronic probes using either integrated μLEDs or passive optical waveguides have been introduced to enable light delivery in deep tissue (Kim et al., 2013; McAlinden et al., 2013; Wu et al., 2013; Hoffman et al., 2015; Schwaerzle et al., 2017; Shin et al., 2017; Noh et al., 2018; Zhao et al., 2018). Probes based on μLED arrays can potentially provide a higher device density for individual optical output ports. Waveguide-based optical neural probes are limited in density because each waveguide needs to be independently routed through the probe shank. Moreover, packaging of commercially-available light sources at the backend further limits the number of independently addressable channels in waveguide-based probes.

To understand the neural basis of brain function and dysfunction, it is essential to perform chronic experiments over a long period of time. Flexible polymer neural probes have recently been shown to reduce chronic damage to the brain by minimizing the tethering force on the tissue and the associated glial scarring (Biran et al., 2007; Lind et al., 2013; Moshayedi et al., 2014; Kozai et al., 2015; Spencer et al., 2017; Wurth et al., 2017). Therefore, such flexible neural probes are potential candidates for long-term chronic neural recording. Adding optical functionality to these polymer neural probes is highly desirable to allow simultaneous stimulation and recording of neural circuits. We have recently demonstrated integration of passive polymer optical waveguides with flexible neural probes to enable simultaneous electrical recording and optical stimulation (Reddy and Chamanzar, 2018a,b). Optoelectronic neural probes consisting of arrays of micro-light-emitting-diodes (μLEDs) operating at optogenetic wavelengths (450–680 nm) with integrated recording electrodes have also been demonstrated by others, but mostly on rigid substrates such as sapphire or silicon (McAlinden et al., 2015; Wu et al., 2015; Shin et al., 2017). Dense integration of μLEDs on flexible substrates remains challenging. Polymer neural probes have been realized based on flip-chip bonding of μLED chips onto a probe shank (Kim et al., 2013; Schwaerzle et al., 2015). This type of integration technique is limited to μLED chips that are separately fabricated and must then be flip-chip bonded onto a flexible substrate. This serial packaging approach of individual μLEDs limits manufacturing throughput. Recently, microfabricated μLEDs were realized on Polyimide or epoxy resin cables by transferring μLEDs from a sapphire substrate using specialized laser processing techniques (Goßler et al., 2014; Klein et al., 2018; Noh et al., 2018). Additionally, 2D arrays of μLEDs have been fabricated on sapphire substrate and bonded to a silicon wafer to take advantage of wafer-scale trace routing on silicon (Klein et al., 2017; Scharf et al., 2018).

Here, we demonstrate fabrication of μLEDs in gallium nitride (GaN)-based heterostructures grown on a silicon wafer, monolithically integrated and encapsulated in a flexible polymer that includes interconnects. The polymer and electrical interconnects are realized on top of the wafer during the fabrication process to encapsulate linear or 2D arrays of GaN μLEDs. The monolithic flexible stack is then released from the silicon substrate at the end of the fabrication process. We demonstrate wafer-scale, high-throughput microfabrication processes to implement μLED neural probes, consisting of monolithically integrated arrays of GaN μLEDs and recording electrodes on a flexible Parylene C polymer substrate. Although GaN-based μLEDs grown on silicon substrates have a low emission efficiency compared to similar devices fabricated on sapphire substrates, the fabrication process is scalable and is based on commonly-used microfabrication techniques. This process allows for a dense integration of optical stimulation and electrical recording functionalities on a unified platform for realizing high-resolution, minimally invasive neural interfaces that can be made available to a wide user base.

## 2. Device Architecture

**Figure 1:**
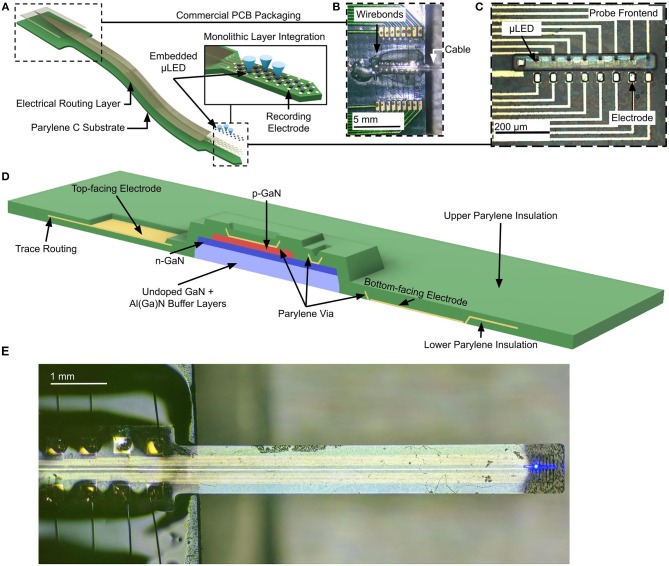
**(A)** Schematic illustration of the probe architecture with a flexible Parylene C cable routing electrical traces from the device backend to the probe active region, containing μLEDs and recording electrodes, **(B)** Packaging of probe backend with consumer PCB via wirebonding, **(C)** Active region of probe, featuring GaN μLED array alongside backside recording electrodes, **(D)** Schematic cross-section of active region showing GaN μLED mesa embedded in Parylene C insulation. The metal trace layer is entirely embedded in Parylene C; front- and backside electrodes are exposed by patterning the top or bottom layer of Parylene C. Traces are connected to GaN *p*-type and *n*-type contacts with vias though the Parylene C insulation. **(E)** Packaged neural probe with linear array active region and 7-mm cable, packaged with a consumer PCB and showing emission from GaN μLED.

Our flexible optoelectronic neural probe architecture allows simultaneous optical and electrical access to the brain, while minimizing tethering force and tissue damage inherent to rigid probe implementations. A schematic of the probe architecture is shown in Figure 1A. The backend of the probe, which stays outside the brain, is designed to interface with the control and signal processing electronic circuit boards (Figure 1B). The frontend of the probe (i.e., the probe shank), shown in Figure 1C, contains dense arrays of collocated μLEDs and recording electrodes. Each layer is lithographically defined, allowing for flexibility in designing the layout of the electrodes and μLEDs along the probe shank.

### 2.1. μLED Light Sources

Arbitrary sizes and shapes of lithographically-defined GaN μLEDs can be realized in our optoelectronic neural probes. As an example, a linear array of μLEDs with a pitch of 60 μm and 30 × 30 μm active area is shown in Figure 1. Two-dimensional arrays with any desired arrangement of light sources and recording electrodes can also be designed in our platform.

The GaN μLED mesas are encapsulated in Parylene C on the top and sides of the mesa, and by insulating undoped GaN and (Al,Ga)N on the bottom surface (Figure 1D). Parylene C is optically transparent at the emission wavelength of our GaN μLEDs (i.e., λ = 445 nm). As opposed to optical neural probes realized on opaque substrates, our μLEDs can emit light from both the front- and backside surfaces for stimulating surrounding neurons on both sides.

### 2.2. Integrated Recording Electrodes

To enable experiments in which neural activity may be monitored and stimulated at the same time, our optoelectronic neural probe platform features collocated recording electrodes and light sources. Recording electrodes and associated interconnects are realized on the same layer as the metal traces for powering the μLEDs. The traces and interconnects are insulated with a second layer of Parylene C. The recording electrodes are then exposed by etching the Parylene C layer covering the electrode sites. The monolithic fabrication process and lithographic patterning of features in each layer of the neural probe enable us to create customizable recording electrode arrangements. In our design, recording electrodes may be fabricated either on the front- or backside of the probe, allowing for directional recording from both sides (Figure 1D). This capability, along with the bidirectional light emission from individual μLEDs, is a unique feature of our design that maximizes the yield of neural recording from volumes of neural tissue on both sides of the probe.

An example of our optoelectronic neural probes is shown in Figure 1E with the active region near the tip of the 7 mm long probe cable.

### 2.3. Monolithic Neural Probes With High Channel Counts

**Figure 2:**
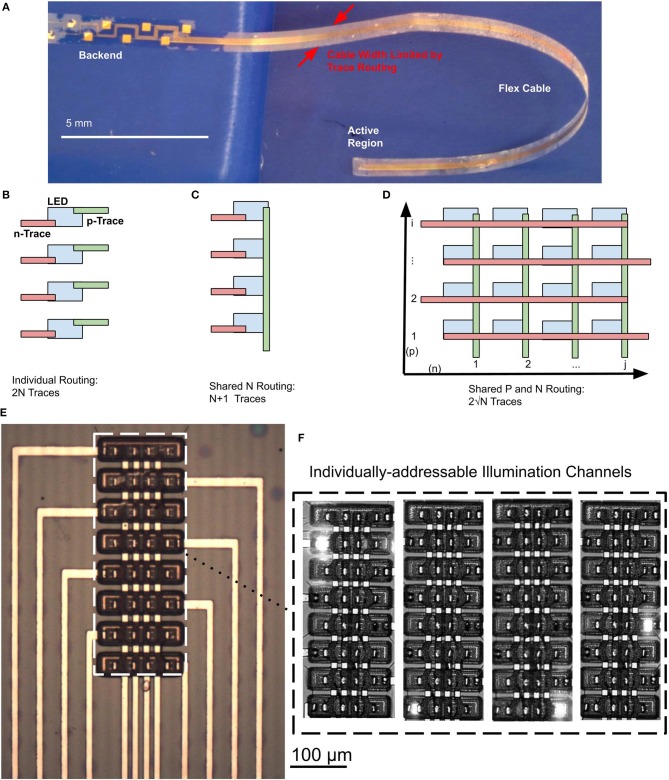
**(A)** Released, flexible 2.1-cm Parylene C cable with embedded μLEDs. Cable dimensions are constrained by the number of traces that needs to be routed in a single layer. **(B)** Trace routing scheme when each μLED has a dedicated electrical trace for the *p*-type and *n*-type contacts, resulting in 2N total traces. **(C)** Trace routing scheme where a linear array of μLEDs share *n*-type contacts, resulting in N + 1 total traces. **(D)** Trace routing scheme where a 2D array of μLEDs share traces for *p*-type and *n*-type contacts, resulting in only 2$\sqrt{N}$ traces. This routing scheme cannot be achieved in a single routing layer, as trace intersections will short the *p*-type and *n*-type traces. **(E)** Microscope image of 2D μLED array. **(F)** Illumination patterns of μLED array showing individual addressability in the 2D routing scheme.

To maximize the read/write bandwidth to and from the brain using a neural probe, a high density of light sources and recording electrodes is essential. In some cases, closely spaced electrodes and light sources may interface with overlapping populations of neurons in the brain, which may result in redundant or overlapping information. However, since the mechanisms of neural encoding at the network-level are not well-understood, it is highly advantageous to stimulate and sample neuronal activity in the brain with high spatio-temporal resolution in multiple distinct regions simultaneously (Buzsáki, 2004; Buzsáki et al., 2015). A rather serious challenge in the design of high-density neural probes is the trade-off between the compactness of the neural probe to minimize damage to tissue, and the need to increase the number of channels in a probe. This trade-off is fundamental and is rooted in the planar fabrication processes, where each recording electrode and each μLED requires a dedicated electrical path that must be routed along the probe cable. As a result of this trade-off, the number of electrical traces scales linearly with the number of additional device channels (optical or electrical), which in the end becomes a major bottleneck to maintaining a narrow width of the probe shank (Figure 2A). Under this circumstance, the width of the probe is determined by the number of traces that must be routed along the cable to the probe shank, i.e., (*n*_*t*_) and the trace pitch (*p*) (Equation 1).

(1) $$\begin{array}{l} W=p\times n_{t}. \end{array}$$

In our current design, we used a conservative trace width of 10 μm for ease of fabrication. However, in our previous work we have demonstrated that traces can be made as small as 300 nm (Chamanzar et al., 2015). In principle, advanced nanofabrication techniques can be employed to implement electrical traces with very narrow widths using high-resolution electron-beam or deep-UV lithography (Shobe et al., 2015; Jun et al., 2017). In general however, specialized lithographic processes are not easily scalable, require dedicated lithography equipment, and are costly to implement. Furthermore, there is a fundamental limitation to the density of traces. As traces become narrower, the electrical resistance increases, although this also depends on the trace thickness. Increasing the trace resistance results in a larger voltage drop on the lines that power μLEDs and also increases the associated thermal noise, decreasing the signal-to-noise ratio for recording electrodes. Therefore, when scaling down the size of traces, resistance must be carefully considered. Reduced spacing between traces also leads to crosstalk between channels. Scaling is only practical to a point through improved lithography resolution. Consequently, an architecture that reduces the number of necessary traces is needed to enable continued future scaling of the number of device channels. In this work, we report an architecture that enables massive scaling of μLEDs on a single neural probe shank, even with large trace widths.

Considering the case of the μLEDs, one notes that each device requires a *p*-type and an *n*-type ohmic contact interconnect. In a naive routing scheme, where each contact trace is routed individually (Figure 2B), the total probe width would be given as

(2) $$\begin{array}{l} W=p\times 2N, \end{array}$$

where *N* is the number of μLED devices. In such a scheme, a probe shank with 200 traces would support a maximum of 100 μLED devices. This scheme can be improved through use of a shared common *n*-type contact in linear arrays (Figure 2C), thus reducing the width of the shank to

(3) $$\begin{array}{l} W=p\times \left(N+1\right), \end{array}$$

since each μLED now requires only one trace for each contact to the *p*-type layer, and all devices share a common trace for the *n*-type contact. In this shared *n*-type contact scheme, a probe shank that can accommodate 200 traces would support a maximum of 199 μLEDs.

To allow for a massive scaling of the number of μLED devices per probe shank, we can design a network of interconnects such that the traces for the *p*-type and the *n*-type contacts are shared among μLEDs in an individually-addressable grid (Figure 2D). Such a scheme would reduce the required probe width to

(4) $$\begin{array}{l} W=p\times 2\sqrt{N} \end{array}$$

in a square device array. In this design, a probe shank capable of accommodating 200 traces would support 10,000 μLEDs, which is a significant improvement over other routing schemes. However, this type of architecture would be precluded by the 2D nature of our planar microfabrication process since perpendicular traces defined in a single layer would cross each other and cause short-circuits. Although multilayer routing schemes are possible, these greatly complicate the fabrication process and reduce yield. Instead, we offer an alternative novel approach to achieving optimal electrical routing without the need for additional trace insulation or routing layers.

Our technique takes advantage of the lithographic definition of each layer of the μLED mesa structure. Since the *p*-type and *n*-type GaN layers are defined in separate lithography steps, they may be formed with arbitrary shapes. In this case, we etch individual *p*-type mesas in a rectangular grid, and etch *n*-type mesas in horizontal strips along the axis of the grid. The routing of each *n*-type “row” of the grid is accomplished by the *n*-type contacts on the GaN mesa itself (Figure 2E), forming a “bridge.” Electrical traces running in vertical “columns” join the *p*-type contacts vertically. The *n*-type layer is separated from the electrical traces by SiO_2_ and Parylene C passivation, providing two separate layers for routing without additional processing steps. This architecture is only possible when the GaN mesas and neural probe cable routing are monolithically integrated, since it requires the geometry in the mesa structures to complement the overall probe routing and architecture.

To demonstrate the feasibility of this monolithically integrated architecture, we have created dense 2D arrays with a pitch of 60 μm along the y-axis and 40 μm along the x-axis. Individual GaN μLEDs have an active area of 22 × 22 μm. Our devices are the first to demonstrate the full 2D implementation of the shared-contact routing scheme. Two-dimensional indexing of μLEDs has been previously suggested by Goßler et al. (2014). The unique feature of our design, which obviates the need for an extra layer of traces in a 2D indexing scheme, is that we use the *n*-type mesa to access μLEDs in each row.

In this architecture, each μLED may be powered and individually addressed by supplying current to the proper trace for the *p*-type contacts and grounding the related trace for *n*-type contacts (Figure 2F). Although arbitrary patterns of μLEDs may not be simultaneously illuminated, arbitrary patterns of neural stimulation are still possible through time-division multiplexing of the μLED channels. In general, GaN optoelectronics can switch at speeds that are several orders of magnitude faster than the relevant time constants of neural activity. For example, GaN LEDs with sizes comparable to our devices, operating at an emission wavelength of 450 nm, can be modulated faster than 300 MHz (Kelly et al., 2012), while the time constants of neuron membrane potentials are at the scale of kHz. Thus, rapid time-division multiplexing between μLED elements would appear as simultaneous stimulation to neurons.

## 3. Methods

### 3.1. Fabrication Methods

**Figure 3:**
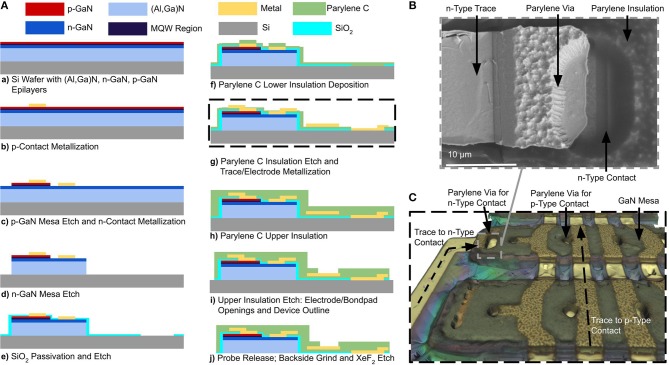
**(A)** Schematic process flow for a μLED neural probe, **(B)** Top-down SEM photomicrograph image of a Parylene C via formed with a sidewall from a photoresist underexposure process. This image is taken perpendicular to the surface; the sloped edge is evident due to the width of the edge. Metal deposited via evaporation is continuous from the top surface of the Parylene C to the μLED contact. **(C)** 3D optical microscope image of GaN mesa structure during fabrication step (g). Traces for *p*-type contacts run orthogonally to the mesas and connect to the *p*-type GaN contacts through Parylene C vias. Traces for the *n*-type contacts are routed through a via on the edge of the GaN mesa and are connected to *n*-type contacts which are patterned along the mesa in the *n*-type GaN layer.

We have designed a scalable, wafer-level fabrication process for high-throughput manufacturing of the optoelectronic neural probes. Fabrication is performed on commercially-available GaN-on-Si epitaxial wafers (Suzhou Innovo China). The substrate is a 2-inch, 1.5-mm thick Si (111) wafer; from the substrate up, the layer structure consists of 900 nm of an (Al,Ga)N buffer layer, 400 nm of an undoped GaN layer, 3,200 nm of an *n*-type doped GaN layer, a 250 nm active region comprised of multiple quantum wells, and a 150 nm *p*-type doped GaN ohmic contact layer. The fabrication process is schematically illustrated in Figure 3A. In the following sections, we will discuss the details of the process.

#### 3.1.1. Lithographically-Defined GaN μLED Mesas

To fabricate GaN μLEDs, the process is as follows: first, metal contacts (17 nm Ni/150 nm Au) are deposited and patterned on the top *p*-type GaN layer of the device structure using an electron beam evaporator (Kurt J. Lesker PVD 75). The contacts are formed using a lift-off process (Figure 3Ab). The *p*-type GaN mesa is then lithographically defined and dry-etched (Figure 3Ac) in chlorine gas chemistry using an inductively-coupled plasma reactive-ion etching (ICP RIE) process (PlasmaTherm Versaline). The etching process was calibrated and timed to etch the *p*-type GaN layer and to stop at the *n*-type layer well below the active multiple quantum well and (Al,Ga)N cladding layers. Metal contacts (17 nm Ni/ 150 nm Au) are then deposited on the exposed *n*-type GaN layer using an electron beam evaporation and lift-off process (Figure 3Ac). The *n*-type layer is then lithographically patterned to define the overall μLED mesa structure. In this process, the *n*-type GaN and (Al,Ga)N layers are etched to the Si substrate (Figure 3Ad) using the chlorine ICP RIE process (PlasmaTherm Versaline). This process requires a thick photoresist layer (10 μm, AZ4620). During the etching process, the backside of the wafer was cooled by the chuck and the etching was cycled in 2-min intervals to prevent substrate heating and reflow of the photoresist.

#### 3.1.2. Parylene C Cables and Vias

To passivate the mesa structures, a 300-nm film of SiO_2_ was deposited on the mesa sidewalls using a plasma-enhanced chemical vapor deposition (PECVD) method in a Trion Orion II PECVD machine (Figure 3Ae). The SiO_2_ was then removed from the μLED contacts and the probe outline using a CHF_3_ RIE process in a PlasmaTherm 790 RIE machine. During this etching step, openings for the backside electrodes are patterned so that the electrode will be exposed when the wafer is released. The SiO_2_ layer under the cable region of the probe was intentionally left intact as an etch-stop layer during the release process. A 5-μm thick layer of Parylene C was then deposited (SCS Labcoter-2) to form the neural probe substrate and lower trace insulation. An adhesion promoter (A174 Silane) was applied prior to the deposition of Parylene C to prevent its premature delamination during processing (Figure 3Af).

The Parylene C deposition is conformal and covers the entire wafer surface. Vias in the Parylene C insulation layer are required for routing traces to the *p*-type and *n*-type ohmic contacts of the μLED, as well as to the backside recording electrodes. Due to the poor selectivity (1:1) of the etching process for photoresist, Parylene C is typically etched using an oxygen plasma RIE process with a hardmask instead of a photoresist mask. Although it provides superior etch selectivity, patterning Parylene C with a hardmask yields steep sidewalls. Such steep sidewalls prevent a continuous metallization layer across the vias, resulting in discontinuous electrical traces and hence poor device yield. To address this issue, we developed an optimized lithography process using a biased mask design with deliberate underexposure to pattern sloped sidewalls in a thick photoresist mask (8 μm), which are then transferred to the Parylene C through oxygen plasma etching (Trion Phantom II RIE). This process creates sloped sidewalls in Parylene C, suitable for realizing vias (Figure 3B). The average sidewall angle was measured to be 70.3° using a combination of top-down scanning electron micrograph (SEM) imaging and step height measurement using a profilometer (KLA Tencor P-15). The sloped sidewalls create process variance in the exact dimensions of the Parylene C openings for contacts, creating larger features than defined on the mask. In practice, this can sometimes lead to shorts between the p-GaN and n-GaN layers when the enlarged contact would overlap with the n-GaN mesa. The precise definition of the contact opening in the SiO_2_ passivation in a separate lithographic step prevents enlarged Parylene C openings from causing shorts between the p-GaN and n-GaN. This technique is highly repeatable and leads to, on average, a device yield of >90% for the continuity of electrical interconnects. During this step, backside recording electrodes are also defined by patterning and etching the lower Parylene C insulation layer all the way to the surface of the Si substrate. The backside electrodes are exposed after the device is released from the silicon substrate.

To further ensure a contiguous electrical connection through vias, a thick metal layer stack (15 nm Pt, 400 nm Au, 15 nm Pt) is deposited using an electron beam evaporation process (Kurt J. Lesker PVD 75) (Figure 3Ag). Platinum is used as the adhesion layer to Parylene C. It also exhibits favorable electrochemical properties for the recording electrodes, including biocompatibility and low electrochemical impedance (Geddes and Roeder, 2003). To perform the lift-off of such a thick metal stack, we used the polydimethylglutarimide-based photoresist-type known as LOR 5B as the lift-off resist. We note that Parylene C has low temperature processing requirements (glass transition temperature 170 °C). However, Parylene C can usually withstand significantly higher temperatures (300 °C) in the absence of oxygen (von Metzen and Stieglitz, 2013). To perform the required 180 °C bake step for LOR 5B resist, an oven with nitrogen atmosphere was used for the bake process. Traces were patterned with a 10 μm width and spacing. The mesa topography and metal interconnects are shown in Figure 3C as a reconstructed 3D microscope image (InfiniteFocus, Alicona Imaging GmbH).

To insulate the metal traces, a second layer of Parylene C is deposited (SCS Labcoter-2) to a thickness of 5 μm (Figure 3Ah). To expose the frontside recording electrodes and bond-pads, the top layer of the Parylene C must be etched. To singulate the neural probe, the top and bottom layers of the Parylene C (which have a total thickness of 10 μm) need to be patterned and etched around the outline of the device. To pattern such a thick layer of Parylene C, we used a 100-nm chromium (Cr) hardmask. The thin film stress in the Cr hardmask layer can sometimes result in the formation of cracks in the Parylene C film. To alleviate this issue, a customized sputtering process (CVC Connexion) was carefully optimized for the deposition of the Cr hardmask layer in order to minimize the thin film stress by adjusting the chamber pressure during the deposition. We found that cracking of the Parylene C was prevented when the Cr thin film stress was less than 1 GPa. Our optimized deposition process yielded a final stress of 600 MPa, providing a safe margin for achieving crack-free Parylene C films. We patterned the Cr hardmask using wet etching (Cr 1020 Etchant, Microchem GmbH). Parylene C was then etched using oxygen plasma (Trion Phantom II). At an etch depth of 5 μm, the bond-pads and frontside electrodes are exposed. The etch is continued to a depth of 10 μm in order to define the probe outline through the top and bottom layers of Parylene C. We observed that over-etching of the electrode and bond-pad sites removes Parylene C residue from the surface. The Cr hardmask is stripped away using a wet etch step in Cr etchant (Cr 1020 Etchant, Microchem GmbH). Following this step, the optoelectronic probes are finally realized on the wafer (Figure 3Ai). The next step is to remove the silicon handle layer and release the probes.

#### 3.1.3. A Grind-and-Etch Release Process to Release Fully Flexible Devices

To release the flexible devices from the Si substrate, the wafer is first ground down from the backside to a thickness of about 100 μm from its initial thickness of 1.5 mm (Grinding Dicing Services, Inc. San Jose, CA). The remaining silicon is then isotropically etched using XeF_2_. The wafer is first mounted on a quartz carrier substrate upside-down using a CrystalBond adhesive and then etched using XeF_2_ (Xactix e2) from the backside. The Parylene C cable is protected by the 300-nm SiO_2_ passivation layer. Once the Si layer is completely removed, the bottom-facing electrodes are exposed. Finally, the probes may be released from the carrier wafer by dissolving the CrystalBond adhesive by soaking the samples for 4 h in Acetone (Figure 3Aj). The SiO_2_ sacrificial layer along the probe cable is removed by dipping the backend in a 49% HF acid solution, leaving only the flexible Parylene C cable.

#### 3.1.4. Released and Packaged Optoelectronic Probes With Electronic Circuit Boards

The released optoelectrodes are packaged with printed circuit boards (PCBs) that connect with a commercial neural recording amplifier through a recording headstage (Intan Inc.) using Omnetics connectors. The PCB adaptor also connects with custom-designed electronic control circuitry for driving individual μLEDs. This electronic control circuitry utilizes a switching network of commercial metal-oxide-semiconductor field-effect transistors to route current to the appropriate traces. A commercial Keithley 2401 Sourcemeter, (Keithley Instruments) with a precise (±100 nA) current control capability, was used as a current source to prevent accidental breakdown of the μLEDs.

To package the released optoelectronic probes with the PCB, we developed a die attach technique using Epotek 301 epoxy to affix the flexible probes to the adapter PCB. We were then able to electrically connect the bond-pads on the backend of the flexible polymer substrate to the PCB using an aluminum wedge-bonder.

### 3.2. Electrophysiology Methods

#### 3.2.1. Brain Slice Preparation

Brain slices recordings were obtained from p22 somatostatin (SST)-Cre/Ai32 mice (SST-Cre, stock: 013044; Ai32(RCL-ChR2(H134R)/YFP), stock: 024109; Jackson Laboratory; Bar Harbor, ME). 350 μm thick coronal slices were cut in ice-cold artificial cerebrospinal fluid (ACSF) containing (in mM): 119 NaCl, 2.5 CaCl_2_, 1 NaH_2_PO_4_, 26.2 NaHCO_3_, 11 glucose on a VT1200S vibrating blade microtome (Leica Biosystems Inc., Buffalo Grove, IL). Slices were recovered for >45 min and maintained at room temperature for 4–6 h under continuous oxygenation (95% O_2_ 5% CO_2_).

#### 3.2.2. Electrophysiology

Visualized juxtacellular and whole cell current clamp recordings were performed on an Olympus BX43 Light Microscope (Tokyo, Japan) using an Axon Instruments MultiClamp 700B microelectrode amplifier (San Jose, USA). Recordings were made from the somata of neocortical ChR2-yellow fluorescent protein (YFP) expressing SST interneurons targeted using FITC optical filter (Olympus-Lifescience, Center Valley, PA) using borosillicate glass electrodes, resistance 6–10 MΩ. Recordings were carried out in modified ACSF containing (in mM): 119 NaCl, 5 KCl, 0.5 MgSO_4_, 1 CaCl_2_, 1 NaH_2_PO_4_, 26.2 NaHCO_3_, 11 glucose. Upon reaching juxtacellular configuration (20–30 MΩ), neurons were stimulated with a 5 pulse train of blue light LED stimulation (10 ms duration, 80 ms interstimulus interval).

#### 3.2.3. Neural Data Analysis

Neural data were acquired using a custom-written IgorPRO software. To eliminate electronic interference from the neural recordings, data were filtered during analysis. Notch filters were applied to eliminate 60 Hz harmonics. Additionally, a bandpass filter from 400 Hz to 5 kHz was applied. The filtering was performed using Python 3.7.2 and Scipy 1.2.1.

## 4. Results

### 4.1. Spectral Intensity of a μLED Suitable for Neural Stimulation

**Figure 4:**
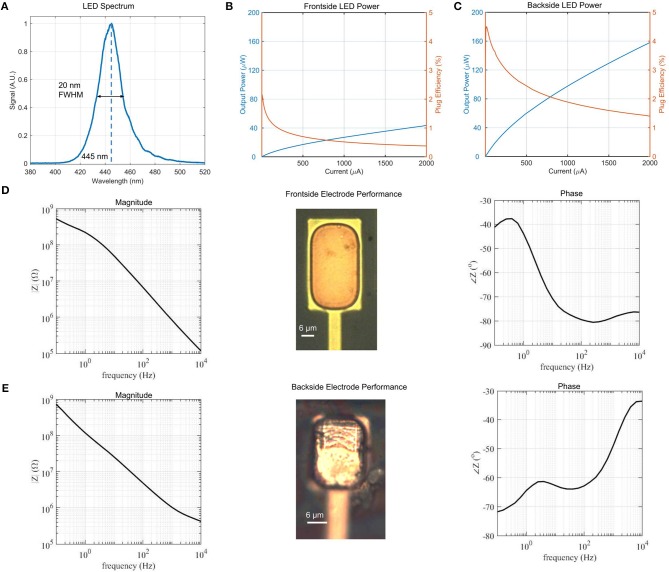
**(A)** Micro-LED spectrum with peak intensity at λ = 445 nm and spectral linewidth of 20 nm FWHM, **(B)** Frontside and **(C)** Backside μLED power and efficiency vs. drive current. Backside emission is approximately 4 times higher due to reflection from the topside metal contacts. Total efficiency of the μLED structure for a given current is the sum of the frontside and backside efficiencies. Peak efficiency is therefore 6.5%. **(D)** Magnitude and phase plots of electrochemical impedance in the relevant frequency range of neural recordings for frontside electrodes. Inset image shows frontside electrode surface. **(E)** Magnitude and phase plots of electrochemical impedance for backside electrodes. Inset image shows backside electrode surface.

The μLED neural probe in this work was designed for spectral overlap with commonly used channelrhodopsin variants that have peak absorption at λ = 450 nm. Our μLED emission peak is at λ = 445 nm, with a narrow spectral bandwidth of 20 nm; this is the full-width-at-half-maximum (FWHM) (Figure 4A). Optical device characterization was performed using a fiber spectrometer (Flame-S, OceanOptics) equipped with an integrating sphere.

The optical power and wall-plug efficiency for individual (22 × 22 μm) μLEDs were measured using a calibrated power meter from the top and bottom surfaces of the device across a range of drive currents (Figures 4B,C). The peak wall plug efficiency of 6.5% agrees with the previously reported results for GaN μLEDs grown on silicon substrate (Wu et al., 2015). The peak efficiency drive current of 8.5 μA corresponds to 540 nW of optical power from the top surface of the probe and 1.3 μW of optical power from the bottom surface. The difference in intensity between top and bottom measurements is due to the reflection of emitted light from the topside U-shaped metal contacts.

### 4.2. Probes With Front- and Backside Recording Electrodes

We characterize the electrodes of our devices using electrochemical impedance spectroscopy (EIS). Measurements were performed in a 3-electrode configuration in potentiostatic mode (PSGSTAT202N, Metrohm Autolab) in a phosphate-buffered saline (PBS) solution. The electrochemical impedance of the electrode was characterized from 0.1 Hz to 10 kHz with a sinusoidal signal whose peak amplitude was 50 mV at an open circuit potential configuration. A silver/silver chloride (Ag/AgCl) reference electrode (MF-2052, BASI Inc.) and a platinum wire counter electrode (MW-1032, BASI Inc.) were used.

Front- and backside electrodes (25 × 35 μm) were fabricated alongside the GaN μLEDs in our architecture. The frontside electrode performance is shown in Figure 4D, while the backside electrode performance is shown in Figure 4E. Both electrodes exhibit a similar electrochemical impedance of ≈1.0 MΩ at 1 kHz, which is typical for electrodes of this size and suitable for neural recording (Ahuja et al., 2008; Neto et al., 2018). The ability to record from both sides of the probe shank, along with the bidirectional emission of the μLED is a unique feature of our design that enables stimulation and recording from a much larger volume compared to the usual single-sided probes.

### 4.3. Thermal and Optical Modeling

#### 4.3.1. Thermal Model

A common concern of implantable μLEDs probes is tissue heating during operation. Due to the low conversion efficiency from electrical to optical power (6.5% peak efficiency demonstrated here), the majority of device power is dissipated as heat into the surrounding medium. We adopt the Pennes bio-heat transfer model in COMSOL Multiphysics (COMSOL Inc.) to demonstrate the threshold of safe operation of the device in tissue (Wu et al., 2015; Dong et al., 2018). The probe geometry was modeled exactly using dimensions from the photomask design files and layer thicknesses measured during the fabrication process. The GaN μLED mesas, metal traces, and Parylene C insulation were included in the model. The relevant material properties are listed in Table 1. Blood perfusion rate, density, and heat capacity are taken from Wu et al. (2015).

**Table 1:** Material properties for thermal simulations.

**Material**	k (W/(m*K))	**ρ (kg/m^**3**^)**	C_**p**_ (J/(kg*K))
Brain Tissue (Dong et al., 2018)	0.6	1,057	3,600
GaN (Dong et al., 2018)	200	6,150	485
Parylene C	0.084	1,110	712
Gold	317	19,300	129

**Figure 5:**
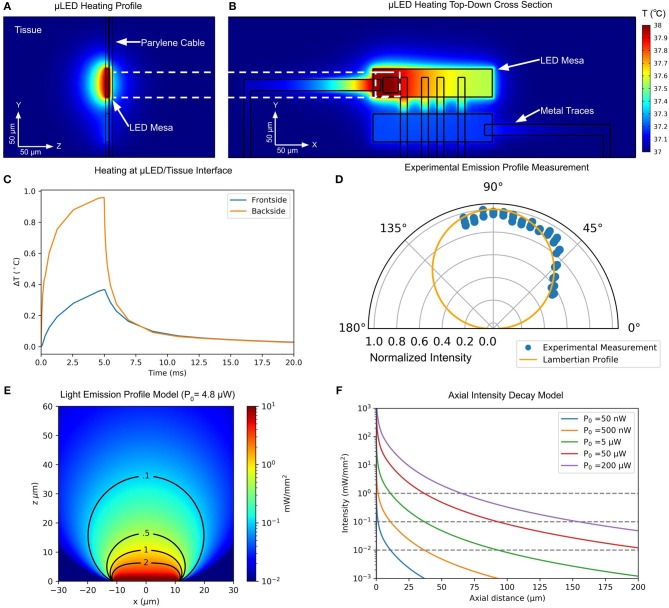
**(A)** Profile of simulated tissue heating using Pennes bio-heat model at 90 μA of μLED drive current. Due to the difference in thermal conductivity between GaN and Parylene C, front- and backside of the probe experience different amounts of tissue heating. **(B)** Top-down cross section of heat spread in μLED device structure at 90 μA of μLED drive current. Thermal conduction in GaN mesa and along metal traces can be observed. **(C)** Tissue heating plot at front- and backside probe/tissue interfaces for 5-ms, 90-μA pulse. **(D)** Experimental measurement of directional μLED intensity profile and comparison to ideal Lambertian source. **(E)** Analytical model of light intensity in tissue from a Lambertian source with 4.8 μW of optical power. **(F)** Analytical model of axial intensity profile in tissue for various optical powers.

Thermal simulations show the effects of local heating in the μLED structure on the surrounding tissue. The front- and backsides of the probe experience different local heating due to the difference in thermal conductivity of the Parylene C insulation on the top surface and the GaN mesa on the bottom. Figure 5A shows the heating profile at the μLED/tissue interface. The tissue in contact with the GaN mesa experiences significantly more heating than the upper Parylene C interface. The heat distribution on a top-view cross-section of the device is shown in Figure 5B. Due to the higher thermal conductivity of the GaN mesa and traces compared to Parylene C and surrounding tissue, heat spreads preferentially throughout the GaN mesa structure and through traces to the adjacent mesas.

To simulate worst-case heating performance, we performed analysis on the forwardmost μLED mesa, which has only one adjacent mesa. The temperature of Parylene C/Tissue and GaN/Tissue interfaces on front- and backside of the probe, directly over the center of the active μLED were monitored in a time-dependent study (Figure 5C). To remain below the standard safety limit of 1 °C temperature increase in tissue, a 5 ms pulse of 90 μA was found to be the limit. This drive current corresponds to a topside optical power of 4.82 μW and a backside power of 14.3 μW. The heat is quickly dissipated in the tissue, and after 10 ms following stimulation offset, the temperature in the surrounding area is less than 0.05 °C above initial temperature (37 °C).

#### 4.3.2. Optical Model

To enable high-resolution interrogation of individual neural circuits, dense two-dimensional arrays of GaN μLEDs may be individually modulated to create independent stimulation voxels in tissue. Analytical and Monte Carlo methods have been developed to describe light scattering in tissue (Wang et al., 1995; Bernstein et al., 2008), with the latter being necessary to accurately model light penetration beyond 200 μm, as light enters the multiple scattering regime (Al-Juboori et al., 2013).

The attenuation of light due to scattering and absorbing media is described via the Kubelka-Monk model, depending on the scattering coefficient, μ_*s*_, and absorption coefficient, μ_*a*_ of tissue. These coefficients vary throughout the brain and also with the wavelength. We adopt values from previously reported measurements at 450 nm, which is the closest wavelength to our emission wavelength (445 nm). The model parameters are $\mu_{s}=19.96{\text{mm}}^{-1}$ (Al-Juboori et al., 2013), and $\mu_{a}=0.14{\text{mm}}^{-1}$ (Yaroslavsky et al., 2002).

We experimentally measured the relative light intensity from multiple angles using a low-NA microscope (NA = 0.10) equipped with a CCD camera on a rotating stage to obtain the μLED emission profile (Figure 5D). Our measured emission profile closely resembled an analytical Lambertian profile (Figure 5D). Therefore, we used an analytical model for the light spread in tissue (Foutz et al., 2012), assuming a Lambertian emission from the μLED.

This model allows for the rapid estimation of penetration depth and light spread for a given optical power. A plot of optical power density spread for 4.8 μW of emitted optical power, which corresponds to frontside emission at our maximum thermally-safe (less than 1 °C) tissue heating threshold, is shown in Figure 5E. A penetration depth of ~40 μm for 0.1 mW/mm^2^ optical power density is observed, with a lateral spread of less than 20 μm. Thus, the stimulation volumes of adjacent high-density μLEDs are independent at this distance.

During chronic implantation, glial encapsulation and reduced local neuron density can hinder the effectiveness of a neural probe, requiring greater depth of penetration to reach healthy cells. Greater depth of penetration can be achieved by increasing the μLED power up to 200 μW. Figure 5F shows the spatial intensity decay of light from the μLED at several ranges of operating power predicted by our model. To stimulate further from the device, multiple μLEDs may be operated as one larger LED to achieve greater overall optical power and penetration depth.

### 4.4. Neuronal Spikes and Synaptic Network Activation Elicited by μLED Illumination

**Figure 6:**
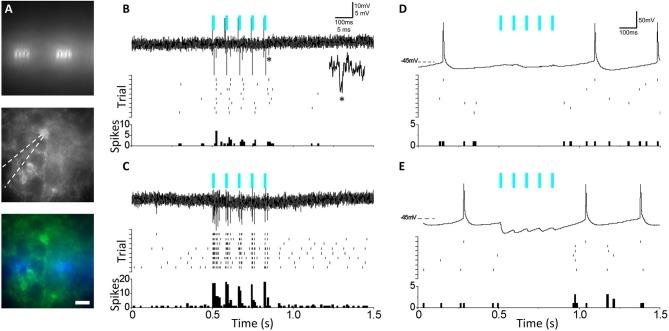
**(A)** Top: Activation of micro-LED (μLED) array embedded beneath recording chamber prior to slice positioning. Center: Targeted recordings from fluorescent ChR2-expressing SST interneurons above array. Bottom: overlay. (Scale = 22 μm). **(B)** Top: Juxtacellular recording showing μLED activation (blue bars) of channelrhodopsin-expressing SST interneuron. *Shown as inset, single μLED evoked spike. Center: Multi-trial spike rasters from the same neuron aligned to μLED activation. Bottom: Peri-stimulus time histograms (PSTH) from the above. **(C)** As in **(B)** but with full-field LED activation of SST interneuron (lower trace) **(D)** Top: As in **(B)** but for whole-cell current clamp recording of neocortical pyramidal neuron showing feedforward inhibition during μLED activation of SST interneurons, with suppression of spontaneous pyramidal neuron firing activity. **(E)** As in **(D)** but using full-field LED activation. IPSPs are larger than with μLED activation and visible as inward currents as V_rest_ >E_Cl_.

To test and validate the efficacy of the μLEDs to elicit evoked activity in channelrhodopsin-expressing neurons, μLED arrays were used to stimulate acute brain slices while simultaneous electrophysiological recordings were conducted. We elected to test μLED array efficacy in driving spikes in a specific subtype of inhibitory neuron, somatostatin-expressing (SST) inhibitory neurons, since these cells typically rest at moderately depolarized potentials (Urban-Ciecko et al., 2018) and it might be easier to drive them to spike in our experimental preparation than pyramidal neurons. The μLED probes were affixed to the bottom of the recording chamber, and brain slices were placed over the probe. SST-targeted recordings were carried out using fluorescence signal from a YFP-tagged channelrhodopsin genetically expressed in these cells, and were selected from the top-surface of the brain slice, above the μLED array (Figure 6A). Upon delivery of a 5-pulse train from μLEDs (10 ms pulse duration, 80 ms inter-pulse interval), ChR2/YFP-expressing neuron showed an increase in evoked spiking, indicating photoactivation (Figure 6B). Onset and offset stimulation artifacts from the μLED were observed with opposite polarity and synchronized with stimulation timings. Due to their highly stereotyped amplitude and waveforms, they are visually distinct from natural spikes. Following μLED illumination, the tissue was subjected to conventional full-field 470 nm light stimulation through a 40x immersion objective lens positioned above the slice using the same pulse train parameters (Figure 6C, top trace). Importantly, full-field optical stimulation elicited more spikes per pulse, likely due to comparatively larger recruitment of the target cell neurites. To test μLED activation of SST-mediated inhibitory synaptic networks, whole-cell current clamp recordings were carried out in neocortical pyramidal neurons (non-ChR2/YFP expressing neurons) from SST-Cre/Ai32 mice. Micro-LED stimulation resulted in inhibition of spontaneous pyramidal neuron firing (Figure 6D, top trace), showing a consistent pause in spiking during μLED pulse train (Figure 6D, mid/bottom). Stimulation was once again changed to conventional full-field 470 nm light stimulation through the 40x objective (Figure 6E, top trace), and a similar synchronized pause in spiking during light pulse train was observed. In contrast to μLED stimulation, conventional full-field illumination elicited distinct optically activated inhibitory postsynaptic potentials (IPSPs), suggesting a more broad activation of local SST interneurons than obtained with the μLED array. Thus, μLED stimulation can both directly activate ChR2-expressing neurons and also indirectly engage feedforward inhibitory networks in acute brain slices.

## 5. Discussion

Our optoelectronic neural probe platform monolithically combines μLEDs and recording electrodes in a flexible Parylene C substrate. The scalable wafer-level fabrication process introduced in this paper greatly increases the process throughput and yield compared to serial packaging processes such as flip-chip bonding. Unlike manual assembly processes, which are limited by the alignment accuracy of the available device bonders and increased fabrication time with the number of devices, lithographic definition of the electrical and photonic devices in our design results in much higher precision, allowing for high-throughput realization of an arbitrary number of devices in parallel. Because we utilize standard microfabrication techniques, our optoelectronic probes can be manufactured with high yield and volume and made available to a wide user base. This approach could conceivably become the standard way for making this type of neural probe for closed-loop interfacing with neural circuits.

Once released, the μLED probe is supported by a flexible Parylene C substrate, which can significantly help with reducing the foreign body response in tissue. However, flexible neural interfaces incur difficulty during implantation since they lack the rigidity to penetrate brain tissue. The flexible neural interfaces need to be temporarily stiffened so that they can be feasibly implanted. This can be carried out by coating the probes with bioresorbable materials such as polyethylene glycol or silk or by attaching them to a stiff shuttle for implantation (Weltman et al., 2016).

The transparency of Parylene C combined with the process-enabled light emission from both facets of the GaN μLEDs, makes it possible to use light in the tissue from the frontside as well as the backside of the device. This new feature means that having only frontside recording electrodes may be inadequate for recording signatures originating from the entire stimulated volume. The design of our platform allows the integration of backside recording electrodes for simultaneous recording and stimulation from both sides of the probe. The emission profiles from the front and back surfaces in this device are asymmetric due to reflections from the metal contacts of the top surface. Symmetric emission profiles could be achieved using thin or transparent contact materials such as indium tin oxide (Kim et al., 2001).

Electrodes on both sides exhibit electrochemical impedances suitable for neural recording. Electrodes are implemented in separate fabrication steps, etching the top Parylene C insulation layer to expose the topside electrodes, and deposition of the metal films for the electrodes through Parylene C vias on the silicon substrate surface, which is eventually etched off during the release process to expose the backside electrodes. These fabrication differences are believed to be responsible for the different phase characteristics of the impedance of the front- and backside electrodes, either because of Parylene C or silicon debris that remains on the surface or a surface modification of the electrode from exposure to the etching gasses.

Furthermore, the monolithic fabrication process described in our work allows for customizable arrangement of recording electrodes and light sources through patterning of individual layers. Unlike traditional flip-chip bonding processes that utilize pre-fabricated μLED chips, we have the flexibility to design complementary structures in various device layers. We leverage this flexibility to enable an individually-addressable 2D grid of devices. By patterning the *n*-type GaN to form connected rows, we are able to use the μLED mesa as an additional layer for electrical routing. This allows creation of arbitrary 2D grids of devices without a second electrical trace routing layer that would complicate the fabrication process. In this particular scheme, the number of traces scales as the square root of the number of devices, as opposed to traditional routing schemes where the number of required electrical traces scales linearly with the number of μLEDs. Therefore, our scheme allows a significant increase in the number of devices that can be realized in a given probe footprint with a single trace routing layer.

Here, we have demonstrated dense arrays of μLEDs with a single n-GaN mesa “bridge” forming the n-layer routing. However, for long linear arrays, the stiffness of the n-GaN layer would quickly compromise the flexibility of the probe. It would be preferable to have many small “floating” GaN mesas at optical stimulation sites connected by polymer cable interconnects. This approach is adopted by Goßler et al. (2014) and Klein et al. (2018), who use two routing layers to separate *p*-type and *n*-type traces. However, multiple routing layers are only necessary where *p*-type and *n*-type traces intersect. We have shown a high-yield method of fabricating Parylene C vias, combined with two-layer routing on the GaN μLED mesa itself. Thus, the 2D grid that we demonstrate could be made sparse, with GaN μLED mesas only at the nodes of the trace matrix, forming a collection of optical stimulators floating in a polymer “web.” Furthermore, additional routing layers may be incorporated in our architecture for linear scaling of cable density, through additional planar layers of Parylene C insulation and vias. We have demonstrated, however, that the fabrication complexity incurred by additional routing layers is not necessary for a 2D routing scheme.

Here, the shank of our demonstration device is very large (570 μm active region), which would be highly damaging during *in-vivo* implantation despite the flexibility of the substrate. This is primarily limited by the conservative trace pitch (16 μm). As previously discussed (Device Architecture Section), the overall size of the device is determined by the lithography resolution and number of required traces. Our 2D routing scheme allows for a significant reduction in the number of traces, and can be combined with high-resolution lithography to create extremely compact, high-density devices.

Our routing scheme also allows for each μLED device in the array to be individually indexed. However, it does not allow for arbitrary patterns of simultaneous illumination. Each μLED device can be indexed in the array by the trace number for the *p*-type and *n*-type contacts: (*p, n*). To activate a given μLED, the indexing *p*-type electrical trace is connected to a current source, and the *n*-type trace to a current sink. Multiple devices may be powered simultaneously by sourcing power to traces for multiple *p*-type and *n*-type contacts. This scheme powers specific sets of devices. For example, devices (1, 1) and (2, 2) may not be powered without activating devices (1, 2) and (2, 1). The set of indexed devices is the cartesian product of the sets of active *p*-type and *n*-type contacts. In this scheme, entire rows of μLEDs may be powered simultaneously.

Although arbitrary patterns of simultaneous illumination are not possible, arbitrary patterns of simultaneous neural stimulation can be achieved through time-division multiplexing. The temporal response of ion channels, which mediates the generation and propagation of action potentials, is generally on the order of milliseconds (kHz); the light sources in our platform, on the other hand, can be modulated at frequency rates in the range of MHz (Kelly et al., 2012). This means time-division multiplexing on these timescales would be indistinguishable to a neuron from continuous illumination. This way, one may generate arbitrary patterns of neural stimulation by rapidly time-division multiplexing sets of μLEDs. Using this method, the average delivered power to neural tissue is reduced since it is split between various μLEDs, providing an ultimate limit to the number of simultaneous optical stimulation sites that can be achieved. Since the maximum output power of our μLEDs is well above the threshold for optical stimulation, still many devices may be multiplexed. Additional research into the behavior of these devices at short pulse durations is needed to ascertain the total number of simultaneous active light sources that could ultimately be possible.

Our devices emit at a center wavelength of 445 nm, which is typical of GaN LEDs widely used in optogenetics. Although this wavelength overlaps well with the peak absorption of channelrhodopsin, shorter wavelengths increase the risk of photochemical damage in tissue, especially in the context of retinal implants (Wu et al., 2006; Soltan et al., 2018). Thus, a longer wavelength which also overlaps with channelrhodopsin absorption, such as 470 nm, may be preferred. The emission wavelength depends on the exact composition of the (Al,Ga)N/GaN or (In,Ga)N/GaN quantum structure in the active region of the device, and emission wavelengths up to 526 nm have been demonstrated (Alhassan et al., 2016). The process described here may be used with any epitaxial GaN-based structure, and thus is generalizable to longer wavelengths with the appropriate composition of the (Al,Ga)N/GaN or (In,Ga)N/GaN active quantum-well region of the GaN-based device structure.

Similarly, the low device conversion efficiency, which raises concerns about tissue heating, is currently limited by the small number of commercial GaN-on-silicon device wafers. In general, GaN-on-sapphire devices are more efficient than ones grown on silicon substrates because of increased lattice mismatch and thermal mismatch issues on silicon. However, silicon-based μLED neural probes offer significant advantages in micromachining such as isotropic XeF_2_ etching, used here, which has been developed for the release of MEMS structures. As a standard microfabrication process, XeF_2_ etching is more widely available and less expensive than laser lift-off (LLO) of GaN on sapphire. Furthermore, the fabrication process discussed in this paper may also be combined with LLO processing of GaN μLEDs fabricated on sapphire substrates.

With our presented conversion efficiencies, the devices may be used in a pulsed mode for high temporal-resolution optogenetic stimulation. We demonstrate a stimulation scheme of 5-ms pulses which leads to the emission of 4.82 μW of optical power from the probe frontside and 14.3 μW from the probe backside, while inducing less than 1 °C temperature increase in surrounding tissue. Such high power may not be necessary *in-vivo*, as Wu et al. have shown robust induced spiking with only 60 nW of optical power (Wu et al., 2015).

The required power depends on the distance between healthy neurons and the optical stimulator after implantation. Acute and chronic damage to tissue by the neural implant reduces neuron density around the implantation site. Although the flexible platform presented here is intended to reduce chronic damage, a “dead zone” around the implant is unavoidable. In this case, higher optical power will be necessary to stimulate neurons, inducing more local heating. However, heating above 1 °C is not a concern in dead tissue. Thus, a higher power stimulation paradigm, where sufficient optical power extends beyond the “dead zone”, but heating above 1 °C is localized, can be envisioned. Since the extent of tissue damage around the neural probe is difficult to predict, the stimulation paradigm for the probe will need to be calibrated after implantation by slowly increasing optical power until robust evoked activity is observed. We showed that our μLEDs, individually driven at 625 μA can stimulate neurons that express channelrhodopsin through a 350-μm thick mouse brain slice.

## 6. Conclusion

To the best of our knowledge, our neural probe platform is the first to monolithically integrate GaN μLEDs and recording electrodes on a flexible polymer substrate using a process that can be achieved in standard microfabrication facilities. This architecture allows the manufacture of extremely high density of μLEDs in 2D arrays. Since each device layer is lithographically defined in the fabrication process, we are able to use the *n*-type GaN mesa as an additional routing layer to create a 2D grid without multiple layers of metal traces. This novel routing scheme enables realization of ultra-compact, high-density, optoelectronic neural probes with correspondingly compact shanks and cables. The overall scheme enables arbitrary patterns of neuronal stimulation. Furthermore, through an optimized wafer-scale fabrication process and post-fabrication packaging, we can achieve a high-throughput manufacturing process to produce a large number of these neural probes. Arbitrary patterns of optical stimulation can be generated using our optoelectrodes. The collocation of recording electrodes and μLEDs enables simultaneous electrophysiology recording and optogenetic stimulation of the brain to study neural circuits with high spatio-temporal resolution.

## Data Availability

The datasets generated for this study will be made available upon reasonable request to the corresponding author.

## Ethics Statement

All experimental procedures involving animals were conducted in accordance with the NIH guidelines and were approved by the Institutional Animal Care and Use Committee at Carnegie Mellon University.

## Author Contributions

JR designed the devices, performed microfabrication, characterization, modeling, and prepared the manuscript. IK designed the devices, performed microfabrication and characterization, and reviewed the manuscript. LS performed electrophysiology and prepared the manuscript. ZA performed EIS measurements and reviewed the manuscript. AB and ET Co-PI, designed and supervised the research, and reviewed the manuscript. MC PI, conceived the idea, designed the research, and prepared the manuscript.

### Conflict of Interest Statement

The authors declare that the research was conducted in the absence of any commercial or financial relationships that could be construed as a potential conflict of interest.
